# Depletion of LncRNA NEAT1 Rescues Mitochondrial Dysfunction Through NEDD4L-Dependent PINK1 Degradation in Animal Models of Alzheimer’s Disease

**DOI:** 10.3389/fncel.2020.00028

**Published:** 2020-02-19

**Authors:** Zhonghua Huang, Jing Zhao, Wei Wang, Jun Zhou, Jie Zhang

**Affiliations:** ^1^Department of Neurology, The Second Xiangya Hospital, Central South University, Changsha, China; ^2^Medical Science Research Center, Xiangya Hospital, Central South University, Changsha, China

**Keywords:** NEAT1, Alzheimer’s disease, PINK1, autophagy, NEDD4L

## Abstract

Alzheimer’s disease (AD) is the most common neurodegenerative disorder and the main cause of dementia among the elderly worldwide. Unfortunately, the mechanism of AD remains unclear, and no effective therapies are available yet. An increasing amount of studies have demonstrated that long non-coding RNAs (LncRNAs) play a notable role in the pathogenesis of plenty of human diseases, and they have served as biomarkers and potential therapeutic targets. However, the function of LncRNAs in AD remains unclear. This study aimed to explore the potential role of LncRNA nuclear enriched abundant transcript 1 (NEAT1) in AD. We found that LncRNA NEAT1 was upregulated in the AD animal models. Furthermore, we demonstrated that NEAT1 could interact with NEDD4L and promote PTEN-induced putative kinase 1 (PINK1)’s ubiquitination and degradation and then impaired PINK1-dependent autophagy. Collectively, the lncRNA NEAT1 promotes the pathogenesis of AD and serves as a promising novel target for pharmacological intervention.

## Introduction

Alzheimer’s disease (AD) is a progressive neurodegenerative disease that slowly destroys memory and thinking skills and eventually leads to the loss of cognitive abilities. AD is characterized by several hallmarks, such as abnormal deposition of Aβ in amyloid plaques, neurofibrillary tangles (NFTs) in the brain, deficits in synaptic function and neuron loss, and abnormal accumulation of Tau and phosphorylated Tau (Yang et al., 2018; Chen and Mobley, 2019). Eventually, the brain tissue of AD patient shrinks significantly as the plaques and tangles spread throughout the brain (Yamazaki et al., 2019). AD has been a major burden to society and the main cause of death in older people. The specific pathogenesis of AD is still unclear, and it is urgent that we carry out in-depth research to block the progression of this disease, improve the survival rates of patients, and provide new countermeasures for clinical treatment. Accumulated evidence has suggested that mitochondrial dysfunction contributes to the pathogenesis of AD and aging-related senile dementia (Moreira et al., 2007; Fukui and Moraes, 2008; Cai and Tammineni, 2016).

The non-coding RNAs account for major proportion of all transcripts according to the project Encyclopedia of DNA Elements (ENCODE) (Consortium, 2012). Long non-coding RNAs (lncRNAs) are greater than 200 nucleotides in length without the protein-coding function (Prinz et al., 2019). Emerging evidence has demonstrated that lncRNAs represent a novel class of pivotal regulators of gene functions and multiple physiological events (Wang et al., 2018, 2019; Yamazaki et al., 2018). They have been found to maintain sub-cellular architecture, stabilize protein complex, and participate in multiple biological processes (Del Vecchio et al., 2018; Gutschner et al., 2018; Liu et al., 2018; Wang et al., 2019; Yan et al., 2019; Yu et al., 2019). First discovered in 2007, nuclear enriched abundant transcript 1 (NEAT1) plays crucial roles both in carcinogenesis (Klec et al., 2019) and non-cancerous diseases, such as neurodegeneration and inflammation (Prinz et al., 2019). NEAT1_1 and NEAT1_2 are two isoforms of NEAT1, which are related lncRNAs that accumulate to high levels in the nucleus (Sasaki et al., 2009). NEAT1 exerts different consequences depending on different downstream mechanisms.

The earliest features of AD have been linked to mitochondrial dysfunction and synaptic damage, including reduced energy production, reactive oxygen species generation, and hypo-metabolisms (Swerdlow, 2012; Fang et al., 2015; Yu et al., 2016). Mitochondrial dysfunctions are critical for the onset and development of AD pathology. Mitophagy is a type of cargo-specific autophagy that is in charge of the clearance of aged or damaged mitochondria. Parkin-mediated mitophagy is a key pathway of mitochondrial quality control. PTEN-induced putative kinase 1 (PINK1) functions as an important regulator in the pathogenesis of AD (Du et al., 2017). PINK1 plays several important roles in the maintenance of mitochondrial integrity and function via mitophagy. A previous study has shown that the expression level of PINK1 is associated with the pathology of AD. Restoring PINK1 can attenuate the Aβ production and amyloid-associated pathology in an AD model via mitophagy (Du et al., 2017).

Mitophagy regulation has emerged as a central problem in the pathogenesis of AD and is a clear therapeutic target for early interference. In this work, we found that the expression level of NEAT1 was upregulated during aging in an APP/PS1 transgenic mouse model. The function and mechanism of NEAT1 in AD were studied. We established a causative link between mitophagy deficits and lncRNA expression in a physiological AD model.

## Materials and Methods

### Reagents

An Aβ ELISA kit was obtained from BIKW Co., Ltd. (Beijing, China). Antibodies against Ubiquitin (Cat.3936), Aβ (Cat.8243), NEDD4L (Cat.5344), PINK1 (Cat.6946), LC3 (Cat.4108), OPTN (Cat.58981), p62 (Cat.885885), and Actin (Cat.3700) were obtained from Cell Signaling Technology (United States). FITC labeled Goat anti Rabbit IgG (Cat.65-6111) and Cyanine5-labeled Goat anti mouse IgG (Cat.M32018) were purchased from ThermoFisher (United States). Antibodies for HA and FLAG were purchased from Sigma (United States). Anti-FLAG M2 magnetic beads (Cat.M8823) and anti-HA magnetic beads (Cat.L-1009) were purchased from Lingyin, Co., Ltd. (Shanghai, China). The magnetic RNA-Protein Pull-Down Kit (Cat.20164) was purchased from ThermoFisher (United States). Real-time PCR kits (Cat.DRR019A) were from Takara (Japan). PINK1, sh-PINK1, NEAT1, and sh-NEAT1 recombinant AAV2 were purchased from Genechem (China). Oligos of siRNAs for PINK1, OPTN, and LC3 were purchased from GemePharma (China).

### Animals

APP/PS1 transgenic mice were purchased from the Laboratory Animal Centre of Xiangya Medical School (Xiangya, China). These animals were bred and housed in standard cages in a climate-controlled room (22 ± 1°C and 50 ± 5% humidity) with 12-h light–dark cycles. The study protocol was approved by the Institutional Animal Care and Use Committee (IACUC) of Central South University (Hunan, China), and all experiments were performed according to the Guidelines of the Association for Assessment and Accreditation of Laboratory Animal Care. The mice were grouped into an untreated control group, an intrahippocampal injection of AAV2-PINK1 (5 × 10^9^ pfu/mouse) group, an intrahippocampal injection of AAV2-shNEAT1 group (5 × 10^9^ pfu/mouse), and an intrahippocampal injection of AAV2-shNEAT1/shPINK1 group (5 × 10^9^ pfu/mouse). Each group contained eight mice. At 60 days post-injection, the mice were subjected to Morris water maze (MWM) testing as previously described (Morris, 1984).

### Cell Culture

HEK293T, SH-SY5Y, and N2A-APPsw cells were cultured in DMEM supplemented with 10% FBS in a 5% CO_2_ atmosphere at 37°C. SH-SY5Y cells were plated in 24-well plates (5 × 10^5^ cells per well) 2 h before the transfection. The cells were transfected with indicated plasmids and cultured for another 24 h, and the cells were then harvested for analysis.

### RNA Isolation and RT-PCR Analysis

The mRNA expression was detected by real-time PCR. Total RNA was extracted from brains using an RNA isolation plus kit according to the manufacturer’s instructions. PCR product formation was monitored continuously using an ABI 7500. Primer sequences for real-time PCR were NEAT1 (mouse), 5′ TGGCTAGCTCAGGGCTTCAG 3′ (sense), 5′-TCTCCTTGCCAAGCTTCCTTC 3′ (anti-sense); GAPDH (Mouse), 5′ GTATTGGGCGCCTGGTCACC 3′ (sense), and 5′ CGCTCCTGGAAGATGGTGATGGT 3′ (anti-sense). Relative gene expression was normalized to GAPDH, and fold change was calculated using the ΔΔCt method.

### RNA Pull-Down Assay

*In vitro* transcription of NEAT1 and biotin labeling were performed to obtain the biotin-labeled NEAT1 RNAs following the manufacturer’s instructions. The biotinylated sense or antisense NEAT1 was incubated with SH-SY5Y cell lysis (with RNAse inhibitor) overnight at 4°C. Streptavidin beads were used to purify the interacting complexes for 1 h at room temperature, followed by mass spectrometry analysis or immunoblotting using a specific antibody to PINK1 or NEDD4L.

### RNA Immunoprecipitation

0.3% Formaldehyde was used to treat SH-SY5Y cells for 10 min at 37°C. After that, the sample was treated with 0.125 M glycine for 5 min at RT. Cells were then washed with PBS buffer three times and centrifuged at 1500 r/min for 2 min. The pellet was then re-suspended in RIPA buffer (1 mM cocktail, 0.1% SDS, 0.5 mM DTT, 50 mM Tris, pH 7.4, 0.5% sodium deoxycholate, 150 mM NaCl, and 1 mM EDTA). The cell lysate was incubated on ice for 30 min with an interval vortex. Antibodies against PINK1, NEDD4L, or IgG control were incubated overnight with the cell lysate at 4°C. Protein G dynabeads were used to recover the RNA/protein complex, and this was followed by washing with RIPA buffer. Finally, the RNA was isolated with Trizol and quantified by real-time PCR.

### Western Blot

Cell lysates and pull-down samples were denatured and loaded to 10% SDS-PAGE. After that, the proteins were transferred onto PVDF membranes, followed by milk blocking and incubation with primary (anti-P62, anti-OPTN, anti-NEDD4L, anti-HA, anti-FLAG, and anti-Ubiquitin) and respective second antibodies.

### Aβ Measurement

Brain homogenates or N2A-APPsw cultured cells were incubated in 5 M guanidine HCl and 50 mM Tris HCl (pH 8.0) overnight. The concentrations of Aβ were analyzed using the commercially ELISA kits following the manufacturer’s instructions. For Aβ immunohistochemistry staining, sections were prepared from 4% paraformaldehyde-fixed brain and stained with Aβ antibody. The positive staining area was determined by image analysis. The investigator was blinded to the mouse genotype.

### ATP Level and Cytochorme C Oxidase Activity

ATP levels were measured using the Bioluminescence Assay Kit (Roche) according to the manufacturer’s instruction as previously described (Yu et al., 2016). Briefly, the mice were anesthetized, and the brains were quickly removed. The hippocampi were homogenized using a lysis buffer followed by centrifuging at 12,000 r/min for 10 min. The ATP levels in subsequent supernatants were measured. The Cytochrome C Oxidase (CcO) activity of mitochondrial fractions was measured as previously described (Fang et al., 2015).

### Statistical Analysis

Each experiment was carried out with triplicate samples. Generally, the experiments were repeated three times. Data are presented as mean ± SE. Difference between two groups was analyzed by respective tests using GraphPad. *p* < 0.05 means statistically significant, ^∗^ means *p* < 0.05, and ^∗∗^ means *p* < 0.01.

## Results

### LncRNA NEAT1 Was Elevated in the AD Mouse Model and Interacted With PINK1 and NEDD4L

**FIGURE 1:**
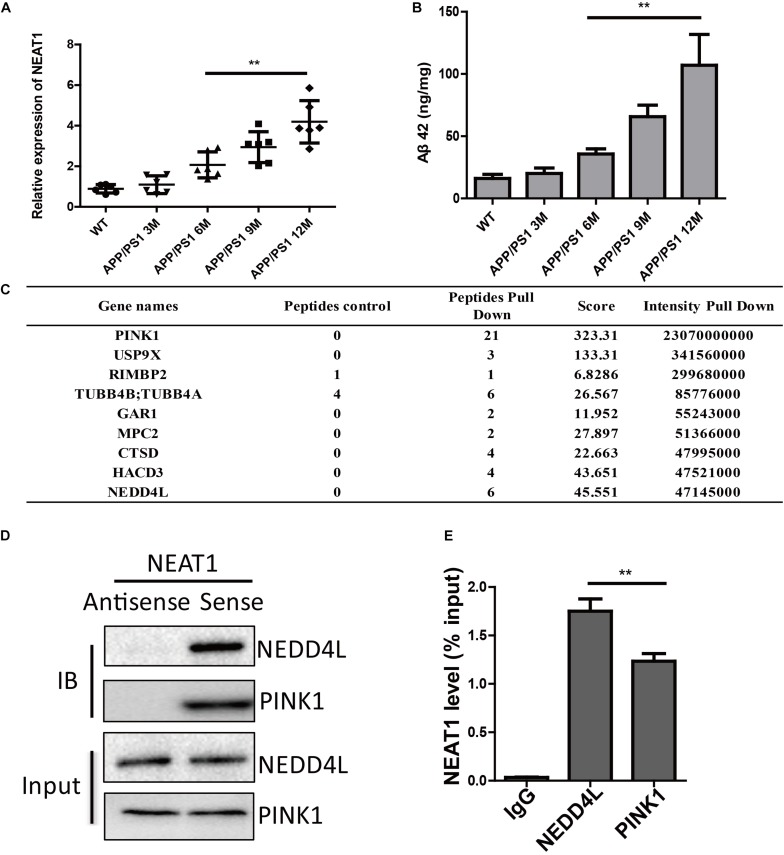
NEAT1 is upregulated in AD mouse models and interacts with PINK1 and NEDD4L. **(A)** Expression of NEAT1 in the hippocampus from APP/PS1 mice of indicated ages was determined by real time PCR. *n* = 5 mice per group. Two-tailed unpaired *t*-test is performed, **p < 0.01. **(B)** Amyloid-β levels in the hippocampus from APP/PS1 mice of indicated ages were measured by ELISA. *n* = 5 mice per group. Bars indicate mean ± SEM, **p < 0.01. **(C)** Proteins bound to biotinylated NEAT1 in SH-SY5Y cells were identified using mass spectrometry and the top candidates were listed. **(D)** Proteins bound to biotinylated NEAT1 were analyzed by western blot using NEDD4L and PINK1 antibodies in SH-SY5Y cells. **(E)** The recovery of NEAT1 is determined by immunoprecipitation with PINK1 or NEDD4L antibody in SH-SY5Y cells. IgG served as negative control. Data are represented as mean ± SEM of three experiments. A two-tailed unpaired *t*-test was performed, and ** means *p* < 0.01 vs. IgG group.

It has been shown that several lncRNAs were dysregulated in the human brain during aging. By investigating differentially expressed lncRNAs in normal and AD brains, we and others have found that the expression of NEAT1 was significantly increased in both aged and AD brains (Cao et al., 2019). To further demonstrate the function of NEAT1 in the AD process, we assayed the expression level of NEAT1 in brains of APP/PS1 mice. NEAT1 was significantly increased in old APP/PS1 mice (over 6 months old) in a time-dependent manner but not in younger littermates (Figure 1A). The expression level of NEAT1 was completely normal in 3-month-old APP/PS1 mice. Similarly, amyloid-β levels showed a similar pattern in brains of APP/PS1 mice (Figure 1B). To better understand the molecular mechanisms of NAET1 in pathology of AD, we labeled the lncRNA NEAT1 with biotin and performed an RNA pull-down assay, followed by mass spectrometry. The top nine NEAT1 interaction proteins were listed (Figure 1C). PINK1 was identified as a potential NEAT1 interaction protein. As PINK1 signaling participated in AD pathogenesis, we performed an independent RNA pull-down assay and western blotting to validate the interaction between NEAT1 and PINK1. Biotin-labeled NEAT1 could interact with PINK1. Interestingly, NEAT1 could interact with the E3 ubiquitin ligase NEDD4L (Figure 1D). Furthermore, the RNA immunoprecipitation experiment using the NEDD4L or PINK1 antibody was carried out to verify the specific interaction (Figure 1E).

### NEDD4L Interacts With PINK1 and Targets PINK1 for Degradation

**FIGURE 2:**
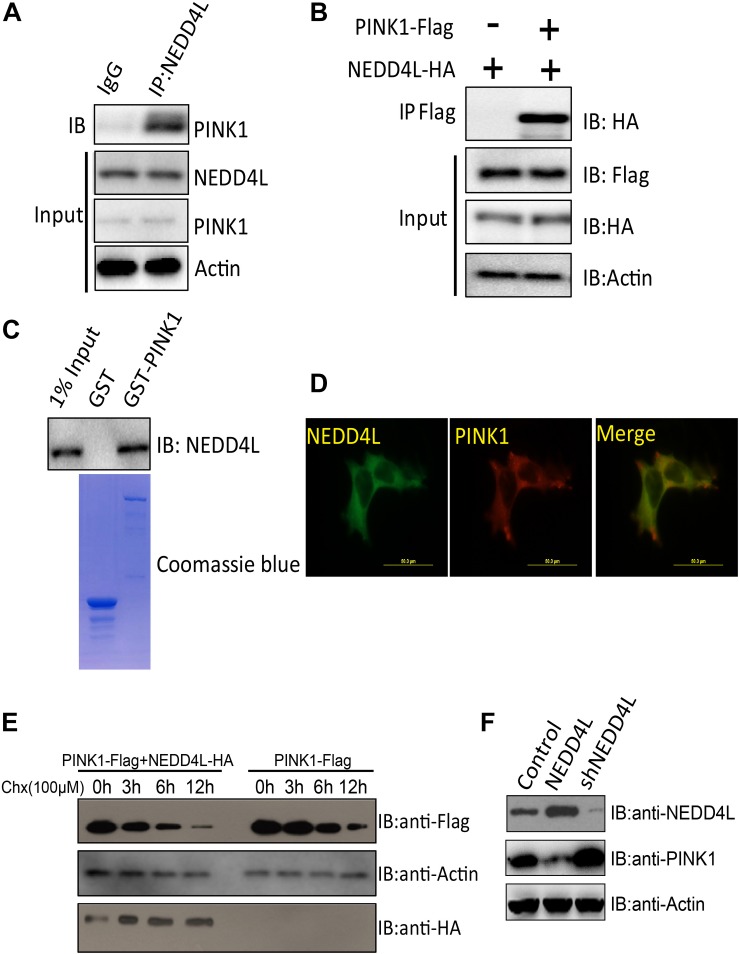
PINK1 was ubiquitinated and degraded by E3 ligase NEDD4L. **(A)** Endogenous NEDD4L interacted with PINK1 in SH-SY5Y cells. SH-SY5Y lysates were immunoprecipitated by an anti-NEDD4L antibody and subjected to western blotting using an anti-PINK1 antibody. **(B)** Flag-tagged PINK1 interacted with NEDD4L-HA. PINK1-Flag and NEDD4L-HA were transfected into SH-SY5Y and co-immunoprecipitation assay was performed. **(C)** GST pull-down assays indicate that recombinant GST-tagged PINK1, but not GST, interacts only with His6-tagged NEDD4L. **(D)** NEDD4L co-localized with PINK1 in SH-SY5Y cells. Immunofluorescence experiments were performed using indicated primary antibodies and respective FITC labeled or Cy5 labeled secondary antibodies. Scale bar: 50 μm. **(E)** NEDD4L-HA and PINK1-Flag expressing SH-SY5Y cells were treated with Cycloheximide (100 μM) for indicated hours. The cell lysates were subjected to western blotting using FLAG or HA antibodies. **(F)** NEDD4L-HA or knockdown plasmids were introduced into SH-SY5Y cells. The cell lysates, 36 h later, were subjected to western blotting.

PINK1 played important roles in orchestrating the parkin-dependent mitophagy. Ubiquitination plays a conserved role in regulating protein turnover. The endogenous co-immunoprecipitation experiment using a NEDD4L antibody demonstrated that NEDD4L could interact with PINK1 in SH-SY5Y cells (Figure 2A). Ectopically expressed FLAG-tagged PINK1 could also be co-immunoprecipitated with NEDD4L (Figure 2B). GST pull-down assays showed that PINK1 directly interacted with NEDD4L *in vitro* (Figure 2C). PINK1 and NEDD4L did colocalize mostly in the extranuclear region of the SH-SY5Y cells (Figure 2D). A Cycloheximide Chase Assay was used to measure the half-life of PINK1. NEDD4L overexpression could exacerbate the degradation of PINK1 in SY-SH5Y cells (Figure 2E). NEDD4L overexpression decreased PINK1 protein levels, and NEDD4L knockdown increased the protein level of PINK1 (Figure 2F). These results indicated that NEDD4L could interact with PINK1 and target PINK1 for degradation.

### NEAT1 Promoted NEDD4L-Mediated PINK1 Degradation and Impaired PINK1-Dependent Autophagy

**FIGURE 3:**
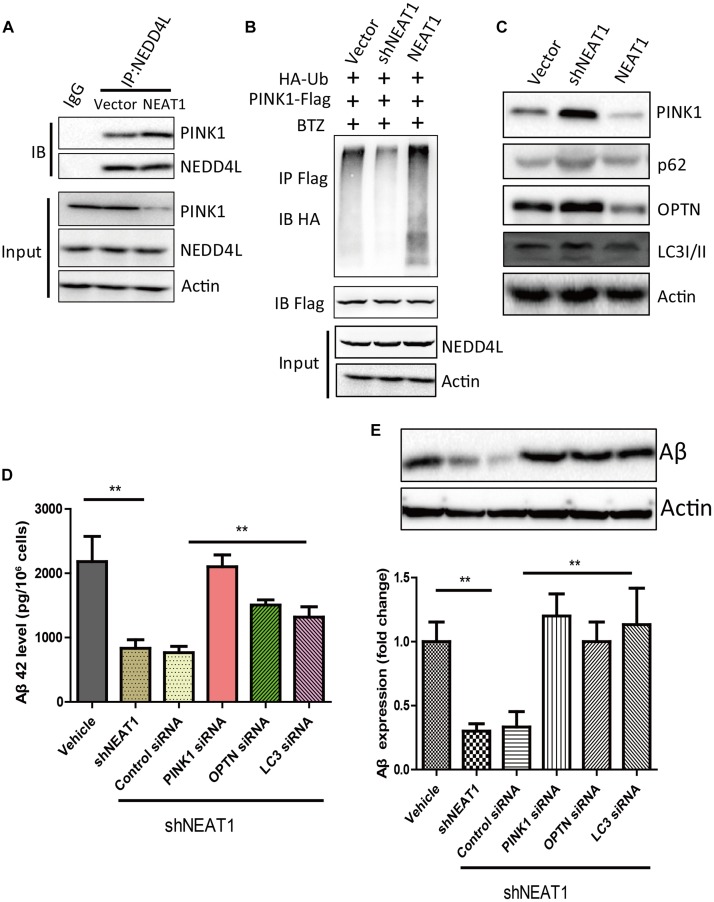
NEAT1 promoted NEDD4L mediated PINK1 degradation and impaired PINK1-dependent autophagy. **(A)** NEAT1 promoted interaction of NEDD4L and PINK1 in SH-SY5Y cells. SH-SY5Y cells were transfected with NEAT1 and cell lysates were immunoprecipitated by anti-NEDD4L antibody and subjected to western blotting using anti-PINK1 antibody. **(B)** NEAT1 regulated ubiquitination of PINK1 in SH-SY5Y cells. NEAT1 or knockdown plasmids accompanied by HA-ubiquitin and PINK1-Flag were delivered into SH-SY5Y cells. The cell lysates were subjected to immunoprecipitation assay followed by western blotting with an anti-Ubiquitin antibody. **(C)** NEAT1 regulated PINK1-dependent autophagy. SH-SY5Y cells transfected with NEAT1 and shNEAT1 were subjected to western blot using PINK1, P62, OPTN, and LC3I/II. **(D)** NEAT1 regulated amyloid-β accumulation via autophagy signaling in N2A-APPsw cells. The levels of amyloid-β42 (Aβ42) in N2A-APPsw cells transduced with lentivirus encoding shNEAT1 and co-transfected with siRNA against PINK1, OPTN, LC3, or control siRNA were measured by amyloid-β ELISA (*n* = 4). **p < 0.01 **(E)** Representative immunoblot bands for amyloid-β proteins in N2A-APPsw cell lysates with above treatment, and β-actin served as a loading control. The bar graph presents the quantification of bands for amyloid-β relative to β-actin. *n* = 4 independent experiments of each group. **p < 0.01.

NEAT1 may be a regulator of mitophagy through interacting with NEDD4L and PINK1. NEAT1 overexpression could promote the interaction of PINK1 with NEDD4L (Figure 3A). NEAT1 overexpression significantly increased the ubiquitination level of PINK1, while NEAT1 knockdown decreased the ubiquitination level of PINK1 (Figure 3B). Moreover, the protein level of PINK1 was increased via NEAT1 knockdown at the same time the autophagy markers were evaluated. Protein levels of P62, OPTN, and LC3 were elevated upon NEAT1 knockdown, whereas these markers were decreased when NEAT1 was overexpressed (Figure 3C). The Aβ level in N2A-APPsw cells was significantly decreased when NEAT1 was knocked down. The Aβ reduction mediated by NEAT1 knockdown depends on PINK1, OPTN, and LC3, as depletion of these autophagy adaptor can reverse the Aβ reduction (Figures 3D,E).

### NEAT1 Regulates Amyloid-β Accumulation in AD Mouse Model

**FIGURE 4:**
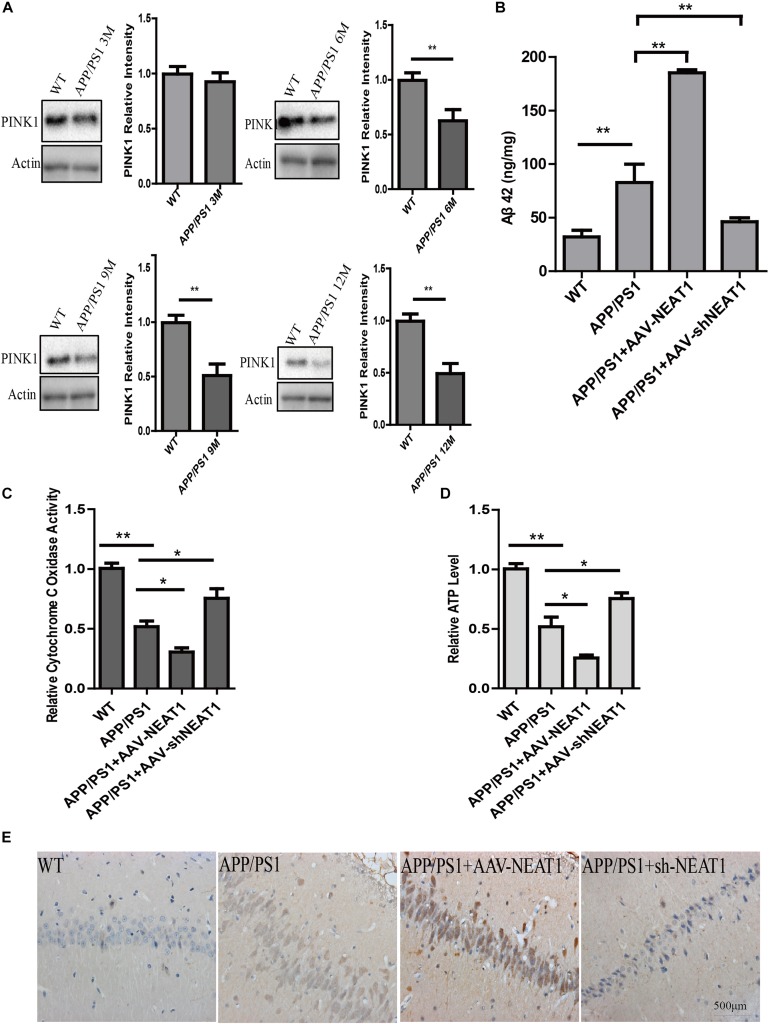
NEAT1 regulates amyloid-β accumulation in AD mouse model. **(A)** Protein level of PINK1 was decreased in the hippocampus of APP/PS1 mice at 6, 9, and 12 months. Hippocampal tissues from APP/PS1 mice of indicated ages were subjected to western blotting using a PINK1 antibody. Representative western blot is shown in the upper panel. **p < 0.01, *n* = 6. **(B)** NEAT1 regulated Aβ levels in AD mouse model. Amyloid-β (Aβ) levels in the hippocampus of the 9-month-old APP/PS1 mice, 2 months post-intrahippocampal injection of AAV2-NEAT1 and AAV2-shNEAT1, were measured by ELISA. **p < 0.01, *n* = 7 mice per group. **(C,D)** Cytochrome C Oxidase activity **(C)** and ATP levels **(D)** in brain tissues of the indicated mice. *n* = 7 mice per group. *p < 0.05, **p < 0.01. **(E)** Representative images of Aβ staining in brain sections of the indicated mice.

The protein level of PINK1 decreased in 6-month-old and 9-month-old mice as well as 12-month-old APP/PS1 mice compared with the normal mice (Figure 4A). AAV-mediated NEAT1 overexpression exacerbated the Aβ production in 9-month-old APP/PS1 mice, whereas the AAV-mediated NEAT1 knockdown inhibited Aβ production (Figure 4B). The enzyme activity of CcO was significantly declined in APP/PS1 hippocampus compared to the normal controls. The activity of CcO in the hippocampus of APP/PS1 mice was further reduced with administration of AAV-NEAT1, whereas CcO activity in the NEAT1 knockdown group was restored to the levels similar to non-transgenic mice (Figure 4C). In parallel, the decrease of ATP level in APP/PS1 hippocampus was reversed via knocking down NEAT1 (Figure 4D). In the brain sections of 9-month-old APP/PS1 mice, the amyloid beta staining shows the similar pattern (Figure 4E).

### NEAT1 Knockdown Ameliorates Cognitive Impairments in AD Mice

**FIGURE 5:**
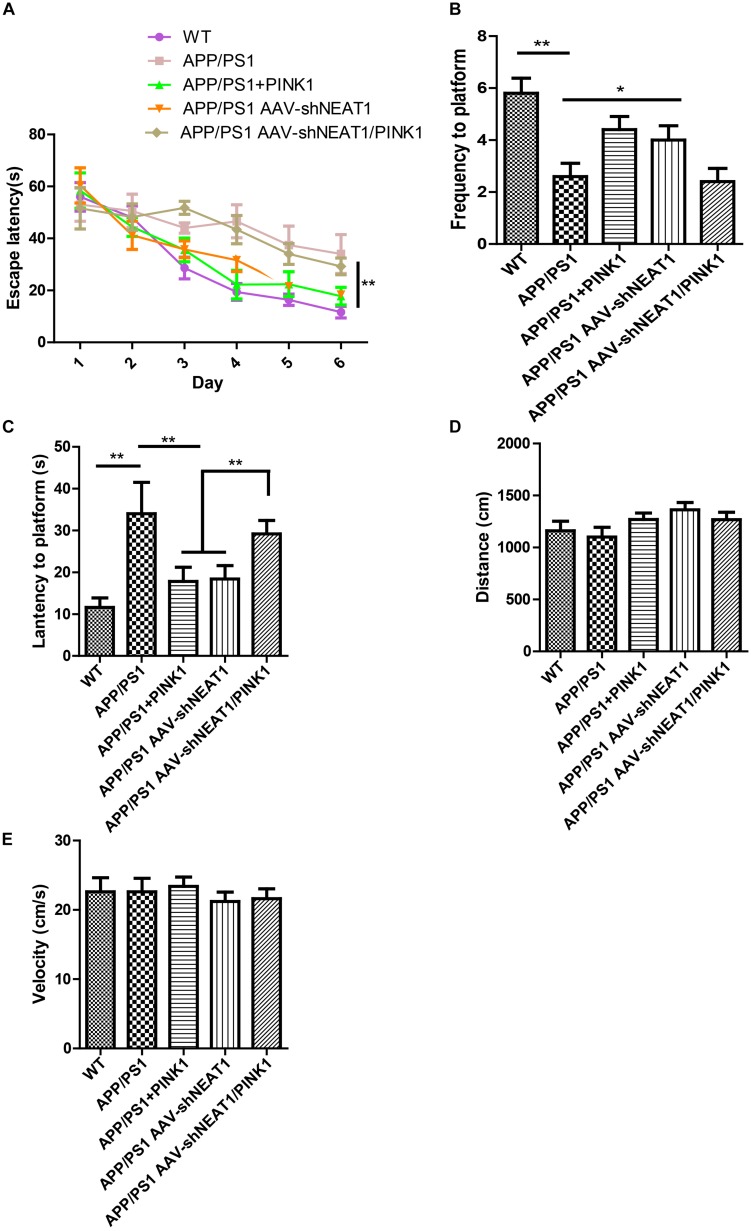
NEAT1 knockdown ameliorates cognitive impairments in AD mice. **(A)** The learning curves of the mice from the different treatment groups (*n* = 8 per group). 9-month-old mice were intrahippocampally injected with AAV2-PINK1, AAV2-shNEAT1, and AAV2-shNEAT1/shPINK1; 2 months later, these mice were subjected to Morris water maze testing. The data are presented as the mean ± SEM, analyzed by two-way ANOVA test followed by Bonferroni, *p < 0.05, **p < 0.01. **(B)** The frequency the target position was passed on day 7 (*n* = 8 per group). The data are presented as the mean ± SEM, analyzed by Kruskal–Wallis test, **p < 0.01. **(C)** The latency to find the platform on day 7 (*n* = 8 per group). The data are presented as the mean ± SEM, analyzed by one-way ANOVA followed by Bonferroni test, **p < 0.01. **(D)** The swimming distance on day 7 (*n* = 8 per group). The data are presented as the mean ± SEM, analyzed by Kruskal–Wallis test. **(E)** The velocity of these mice on day 7 (*n* = 8 per group). The data are presented as the mean ± SEM, analyzed by a Kruskal–Wallis test.

To evaluate the effects of NEAT1 on the pathophysiology of AD, spatial memory was assessed using the MWM. A two-way ANOVA was performed to analyze the latency to platform. Compared with their wild-type littermates, the 12-month-old APP/PS1 mice exhibited a significantly slower learning rate, indicating cognitive deficits in these mice [day 3, *P* < 0.001]. Notably, PINK1 overexpression or NEAT1 depletion significantly ameliorated learning and memory impairment in these APP/PS1 mice (Figure 5A). Compared with the normal mice, the PINK1 overexpression or NEAT1 depletion APP/PS1 mice crossed the platform area more often (Figure 5B) and took less time to reach the position of platform (Figure 5C). PINK1 depletion resulted in the spatial memory defect in the NEAT1-injected APP/PS1 mice, suggesting that the function of NEAT1 was PINK1 dependent (Figures 5A,C). There was no detectable difference in swimming distance or velocity between different groups, indicating that NEAT1 or PINK1 had little influence on the motor ability or motivation of these mice (Figures 5D,E). Together, our data indicate that the depletion of NEAT1 ameliorates cognitive deficits in AD model mice.

## Discussion

**FIGURE 6:**
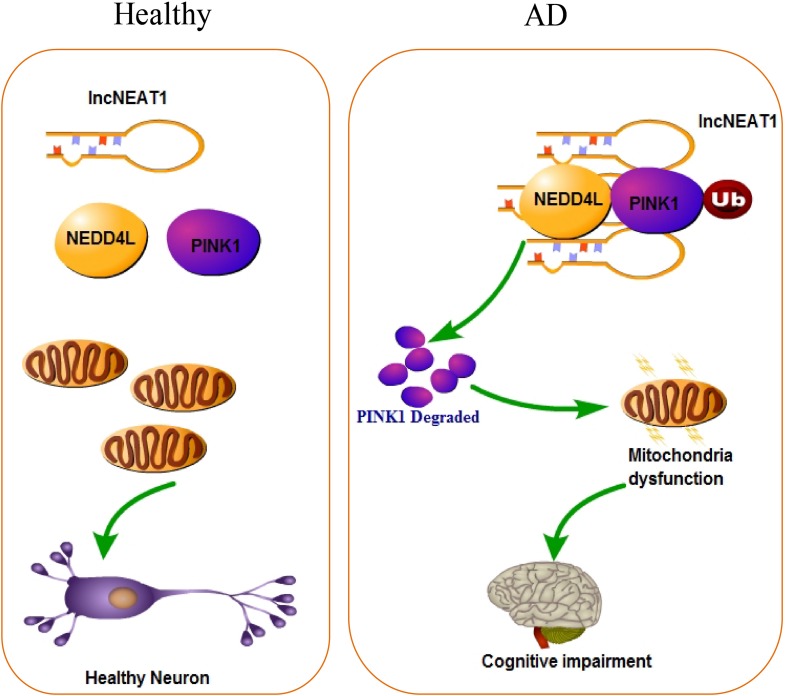
Working model of NEAT1 in healthy neurons and AD.

Alzheimer’s disease has been known to science for more than 100 years, but the pathological mechanism is still unclear. Up to now, there are more than 30 hypotheses trying to explain the pathogenesis of AD, including—but not limited to—the well-known Aβ deposition, NFTs, neuroinflammation, and craniocerebral trauma. With the rapid development of life science in recent years, the industry’s in-depth understanding of the complex mechanism of AD has continuously expanded. In the present study, we found that lncRNA NEAT1 was upregulated in APP/PS1 mice that were over 6 months old in a time-dependent manner but not in younger littermates with the same trends of Aβ levels in the hippocampus. In the meantime, using an RNA pull-down assay, PINK1 and E3 ubiquitin ligase NEDD4L were identified as potential NEAT1 interaction proteins. NEAT1 could regulate the interaction between PINK1 and NEDD4L. Our data suggested a working model of NEAT1, illustrated in Figure 6, in which the upregulated NEAT1 promoted the ubiquitination and degradation of PINK1, which finally inhibited the autophagy signaling and gave rise to the amyloid accumulation and cognitive decline.

The majority of human transcripts are non-coding RNAs, which are composed of microRNAs (miRNAs), piRNAs, siRNAs, cirRNAs, and the lncRNAs (Klec et al., 2019). Despite the non-coding property, lncRNAs are key functional regulators in cellular processes. NEAT1 was part of paraspeckles and was discovered in 2007. Dysregulation of lncRNA NEAT1 was found in an increasing number of diseases. In many cases, NEAT1 functions as a competing endogenous RNA (ceRNA) that sponges miRNA. The transcription or translation of the miRNA downstream targets were hampered and ultimately contribute to diseases. This oncogenic role of NEAT1 in cancers suggests that it may be a promising biomarker and also a candidate therapeutic target pending the completion of further studies into the underlying mechanisms (Carpenter et al., 2013; Ii et al., 2014; Liu et al., 2015). Recent studies have indicated that lncRNAs contribute to the development and progression of several brain disorders, including AD (Faghihi et al., 2008; Cao et al., 2019; Yi et al., 2019). Several differentially expressed lncRNAs were identified in human AD brains, including but not limited to n341006, LINC01094, AD-linc1, and NEAT1 (Magistri et al., 2015; Zhou and Xu, 2015; Cao et al., 2019; Zhou et al., 2019). We found that lncRNA NEAT1 was elevated during aging in the APP/PS1 mouse model. A recent study indicated that NEAT1 was significantly elevated in the temporal cortex and hippocampus of AD patients (Spreafico et al., 2018), implying that NEAT1 was a biomarker for AD diagnosis. They further demonstrated that NEAT1 could negatively regulate the expression of cyclin-dependent kinase 5 regulatory subunit 1, an AD related gene, and possibly played a protective role against neuronal death.

NEDD4L is short for neuronal precursor cell-expressed developmentally downregulated 4-like. NEDD4L is a highly conserved eukaryotic E3 ubiquitin ligase, which belongs to the HECT family and is widely expressed in adult mouse brains (Yanpallewar et al., 2016; Zhang et al., 2017). Previous studies showed that NEDD4L regulated plenty of ion channels, including chloride channels, voltage-gated Na^+^ channels, voltage-gated K^+^ channels, and glutamate transporters (Abriel and Staub, 2005; Bongiorno et al., 2011; Goel et al., 2015). In Parkinson’s disease (PD) model, NEDD4L promoted the ubiquitination of glutamate transporters *in vitro* and *in vivo*. NEDD4L knockdown could increase the protein level of glutamate transporters and rescue the motor deficits and tyrosine hydroxylase expression in PD mice, suggesting that NEDD4L could be a potential therapeutic target for the treatment of PD (Zhang et al., 2017). In our study, we demonstrated that NEDD4L was an E3 ubiquitin ligase for PINK1. NEAT1 could increase the interaction of NEDD4L and PINK1, thus promoting the ubiquitination and ubiquitin-dependent proteolysis of PINK1. Our results demonstrated that lncRNA NEAT1 provided a higher degree of complexity to the control of pathogenesis of AD.

Mitochondria provide an essential source of energy and play an important role during development and in AD progression. Cumulative evidence illustrates the importance of mitochondrial quality control in brain function during aging and cognitive deficit (Flannery and Trushina, 2019). Mitophagy was an important regulator of mitochondrial quality control, both in physiological and pathophysiological conditions. It is increasingly recognized that mitophagy is critical for mitochondrial turnover, especially the damaged mitochondrial clearance. PINK1-mediated autophagy signaling promoted the damaged mitochondrial clearance and contributed to amyloid-β degradation and clearance. Depletion of NEAT1 upregulated expression levels of autophagy receptors, such as OPTN, P62, and LC3, in amyloid-β enriched cells. Depletion of NEAT1 showed a reduction in amyloid-β accumulation, while knockdown of PINK1, OPTN, or LC3 abolishes this protective effect, indicating that NEAT1 regulated amyloid pathology via PINK1 activated mitophagy.

Alzheimer’s disease represents the leading cause of death in old people across the world. It is invaluable to understand the function of lncRNA NEAT1 in regulating mitophagy which protects brain from cognitive decline. Constitutive mitophagy is a homeostatic mechanism for maintaining mitochondrial quality and global mitochondrial function not only in the brain but also in other tissues. Cumulative data have demonstrated the important role of mitophagy in neuronal degeneration. This study has produced critical insight into the role of lncRNA NEAT1 in AD pathological conditions, and will fundamentally advance our understanding of the interaction between lncRNA and mitophagy in AD. Furthermore, NEAT1 may be a useful diagnosis biomarker for AD. Our study may provide the basis for novel therapeutic approaches in AD and a wide variety of aging and dementia.

## Data Availability Statement

All datasets generated for this study are included in the article/supplementary material.

## Ethics Statement

This study was approved by the Institutional Animal Care and Use Committee (IACUC) of Central South University (Hunan, China), and the experiments were conducted according to the Guidelines of the American Physiological Society.

## Author Contributions

JieZ conceived and designed the study. ZH, JinZ, and WW carried out the experiments and collected all data. ZH, JieZ, and JunZ drafted the manuscript. All authors read and approved the final manuscript.

## Conflict of Interest

The authors declare that the research was conducted in the absence of any commercial or financial relationships that could be construed as a potential conflict of interest.
